# High-Performance Copper Oxide Visible-Light Photodetector via Grain-Structure Model

**DOI:** 10.1038/s41598-019-43667-9

**Published:** 2019-05-14

**Authors:** Hyeon-Joo Song, Min-Ho Seo, Kwang-Wook Choi, Min-Seung Jo, Jae-Young Yoo, Jun-Bo Yoon

**Affiliations:** 1 School of Electrical Engineering, Korea Advanced Institute of Science and Technology (KAIST), 291 Daehak-ro, Yuseong-gu, Daejeon 34141 Republic of Korea; 2 Information & Electronics Research Institute Korea Advanced Institute of Science and Technology (KAIST), 291, Daehak-ro, Yuseong-gu, Daejeon 34141 Republic of Korea

**Keywords:** Optical sensors, Materials for optics

## Abstract

Recently, copper oxide (CuO)-based visible-light photodetectors have attracted great interest due to their narrow bandgap (1.2 eV), low cost, and ease of fabrication. However, there has been insufficient theoretical analysis and study of CuO-based photodetectors, resulting in inferior performance in terms of responsivity, detectivity, and response speed. This work develops a method to enhance the performance of CuO photodetectors by engineering a grain structure based on a newly-developed theoretical model. In the developed theoretical grain-structure model, the grain size and the connections between grains are considered because they can strongly affect the optoelectronic characteristics of CuO photodetectors. Based upon the proposed model, the engineered CuO device achieves enhanced optoelectronic performance. The engineered device shows high responsivity of 15.3 A/W and detectivity of 1.08 × 10^11^ Jones, which are 18 and 50 times better than those of the unoptimized device, and also shows fast rising and decaying response speeds of 0.682 s and 1.77 s, respectively. In addition, the proposed method is suitable for the mass-production of performance-enhanced, reliable photodetectors. By using a conventional semiconductor fabrication process, a photodetector-array is demonstrated on a 4-inch wafer. The fabricated devices show uniform, high, and stable optoelectronic performance for a month.

## Introduction

Visible-light photodetectors are essential for various optoelectronic applications, such as imaging, environmental research, chemical analysis, and optical communications^1–3^. Conventionally, visible-light photodetectors have been demonstrated with hard film-type silicon (Si), which has low noise and high detectivity^4^. However, the conventional film-type Si photodetectors are undesirable for recently emerging flexible and transparent electronics and internet of things (IoT) applications, because of their bulky and brittle material properties and cost-inefficiency^5^. For these reasons, recent studies have been conducted to develop simple and cost effective high-performance photodetectors based on material and structural approaches. For example, metal oxides^6–12^, transition metal dichalcogenides (TMD)^3,5,13–15^, and perovskites^16–20^, have been developed for the light-absorbing material of photodetectors to achieve high sensitivity and fast response speed. Also, mechanical flexibility and enhanced light absorption of sensing materials have been developed by introducing zero-dimensional (0-D) (quantum dots), 1-D (nanowires, nanotubes, nanorods), and 2-D (nanosheets, monolayers, nanofilms) nanostructures^1,8–12,18,19,21–26^.

Among various materials, copper oxide (CuO), is highly suitable for high-performance and cost-effective visible-light photodetectors because of its narrow band gap of 1.2 eV, abundance, and mechanical stability based on its atomic structures^27,28^. In particular, CuO has received considerable attention in both academic and industrial fields because of not only its versatile material properties, but also its fabrication compatibility with conventional semiconductor technology^29–37^. However, the research on CuO-based photodetectors is at an early stage, so there is still a lack of deep understanding. Previous studies on CuO-based photodetectors are about simple material quality^34,35,37^ and shape changes^29–32,36^; thus, rigorous study on the optoelectronic characteristics of CuO and significant performance enhancement in device-scale, such as responsivity, detectivity, and response time is necessary.

In this work, we investigated a method to demonstrate a performance-enhanced CuO photodetector supported by a newly-developed grain-structure model. We first theoretically developed a grain-structure model based on conventional photo-carrier generation/recombination and oxygen adsorption/desorption mechanisms. We also studied methods to improve the optoelectronic characteristics of the CuO photodetector based on the developed grain-structure model. By controlling the grain size and contacting size between the CuO grains, the dark current and the photocurrent generation can be engineered, resulting in enhanced optoelectronic characteristics of CuO, such as responsivity, detectivity, and response time. We also experimentally confirmed that the optoelectronic characteristics of CuO photodetectors can be controlled by application of the proposed model. By fabricating various CuO nanofilms that have various grain sizes and contacting sizes of grains using a metallic copper (Cu) oxidation method, larger grain and smaller contacting-size-based CuO photodetectors were demonstrated; they successfully exhibited reduced dark current as well as enhanced photocurrent and response speed, which are highly consistent with the proposed grain-structure model. As a result, the film having the largest grain size and smallest neck size successfully showed the most performance enhancement. Finally, we further confirmed that the proposed concept is highly suitable for industrial-level large-area fabrication. Using a conventional semiconductor process, the CuO photodetectors that showed the most performance enhancement were fabricated on a 4-inch wafer, and we confirmed their fabrication and performance reliability.

## Proposed Model and Principle

**Figure 1:**
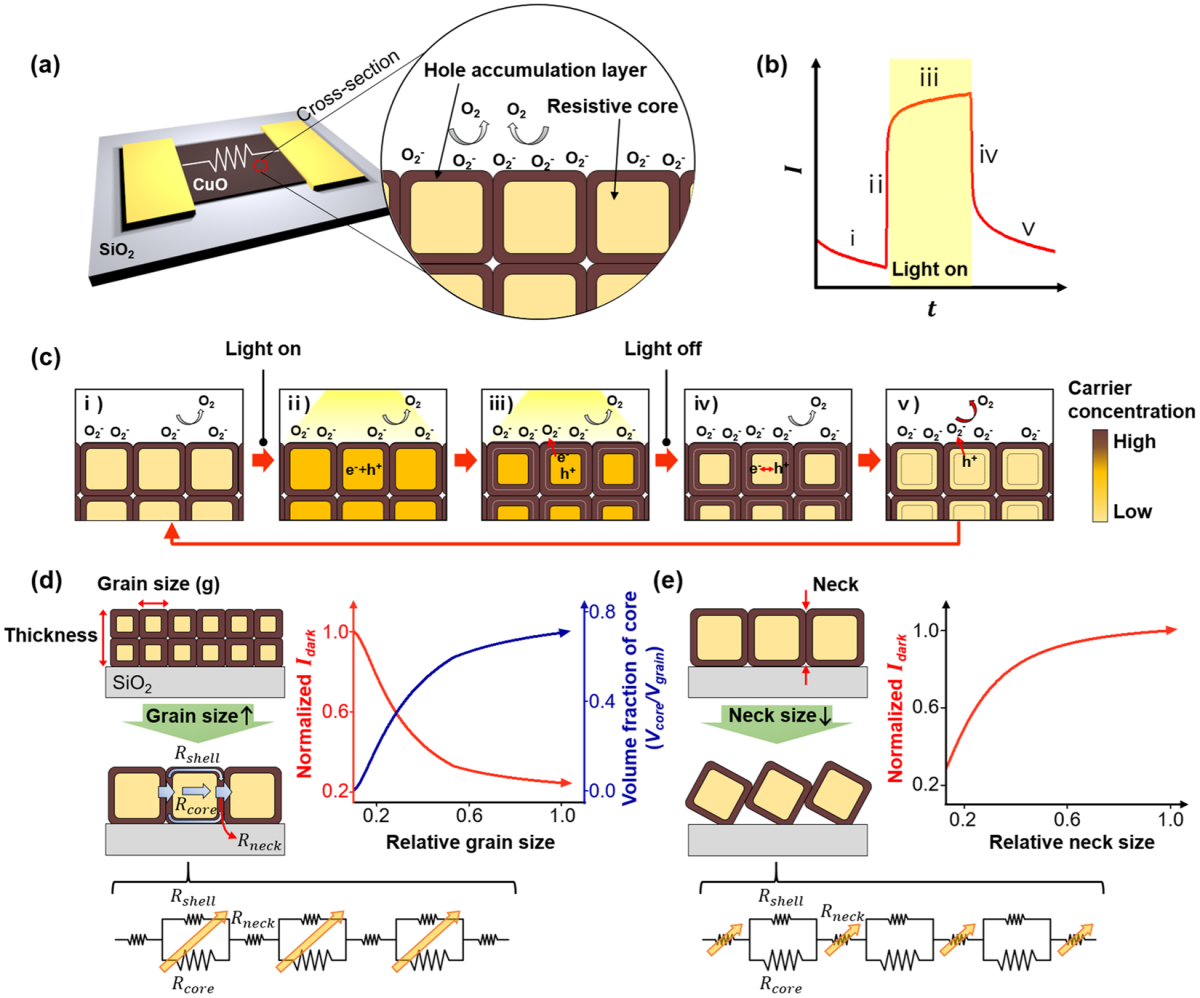
Overview of the proposed CuO photodetector model. (**a**) Schematic illustration of a polycrystalline p-type CuO nanofilm photodetector and its cross-sectional view. (**b**) Typical time-dependent current curve of the photodetector under light illumination. (**c**) Cross-sectional schematic views of the photodetecting process in the CuO photodetector. Schematic description of the effect of (**d**) the grain size and (**e**) the neck size on a dark current.

The metal-oxide-based photodetector is basically operated by the conventional photo-carrier generation/recombination and oxygen adsorption/desorption mechanisms^30,38–41^. Figure 1(a–c) shows the proposed model in detail. In case of CuO film, a p-type semiconductor, the grain can consist of an electrically resistive core and an electrically conductive layer formed by hole accumulation on the surface due to the adsorbed oxygen molecules in an air ambient (Fig. 1(a))^30,42,43^. As light illuminates, a photocurrent is generated abruptly by the large and fast generation of electron-hole pairs [hv → e^−^ + h^+^] (Fig. 1(b,c,ii)). This process mainly occurs at the resistive core rather than the hole accumulation layer where the carrier recombination probability is relatively high because of high carrier concentration. During light illumination, photo-generated electrons participate in oxygen adsorption at the hole accumulation layer [O_2_ + e^−^ → O_2_^−^], resulting in the prolonged lifetime of unpaired holes, while increasing the hole accumulation layer depth. This surface oxygen reaction occurs slowly, leading to a gradual increase in photocurrent (Fig. 1(b,c,iii)). Once the light is turned off, electrons and holes recombine with each other quickly, and then the photocurrent falls abruptly (Fig. 1(b,c,iv)). Additionally, remaining holes discharge ionized oxygen ions [h^+^ + O_2_^−^ → O_2_], resulting in shrinkage of the hole accumulation layer, which contributes to slow decay in the photocurrent (Fig. 1(b,c,v)). From the proposed model, we can expect that the dark current, photocurrent, and response time are strongly affected by the grain structure. Based on this grain-structure model, we devised a method to enhance the optoelectronic characteristics of the CuO nanofilm (Fig. 1(d,e)). The key idea is that the dark current (*I*_*dark*_), which is one of the most important factor for the responsivity and detectivity, can be changed by variation of the grain structures, such as the volume fraction of the resistive core to the hole-accumulated shell and the contacting size between adjacent grains (defined as the neck size). (i) Changes in *I*_*dark*_ according to the grain size (volume fraction of the core to the shell) – When the volume fraction of the total core is increased due to increased grain size (volume) under the condition of a constant thickness (upper-left panel in Fig. 1(d)), *I*_*dark*_ can be decreased by increasing the electrical resistance of the nanofilm because the electrical resistance of the increased volume fraction of the core is much higher than that of the shell (lower panel in Fig. 1(d)). (ii) Changes in *I*_*dark*_ according to the neck size – The *I*_*dark*_ can be decreased by decreasing the neck size because the contact resistance between CuO grains is dominated by the necks of adjacent grains (upper-left and lower panels in Fig. 1(e)), and the resistance is increased by decreasing the neck size. To specifically understand *I*_*dark*_ variation based on the grain-structure model, we analytically calculated *I*_*dark*_. It should be noted that, for simplicity in modeling, we assume that all grains have cubic or cuboid structures and that their surfaces are in contact with adjacent ones. Moreover, the neck is simplified with the configuration of cubic grains attached to each other by the edge parts of their surfaces (Fig. 1(e))^44^. The modeling and analytic calculation are presented in detail in Fig. S1 and concept and calculation method in Supplementary Information. From the proposed model, the *I*_*dark*_ can be calculated to show its changes with respect to the grain and neck sizes (upper-right panels in Fig. 1(d,e)). From the analytical calculation, we confirmed that the increased grain size and decreased neck size of CuO are very important for performance enhancement in CuO photodetectors. Interestingly, the relationship between the grain size (grain boundary) and the optical performances in CuO (p-type) photodetectors that we found in this work is exactly opposite to that of ZnO (n-type) photodetectors, because an electrically conductive surface and non-conductive core are formed in the p-type material, whereas an electrically insulating surface and conductive core are formed in the n-type material^45^. Therefore, a larger grain-size is beneficial to the p-type material, however, it is not to the n-type material.

## Sample Preparation

**Figure 2:**
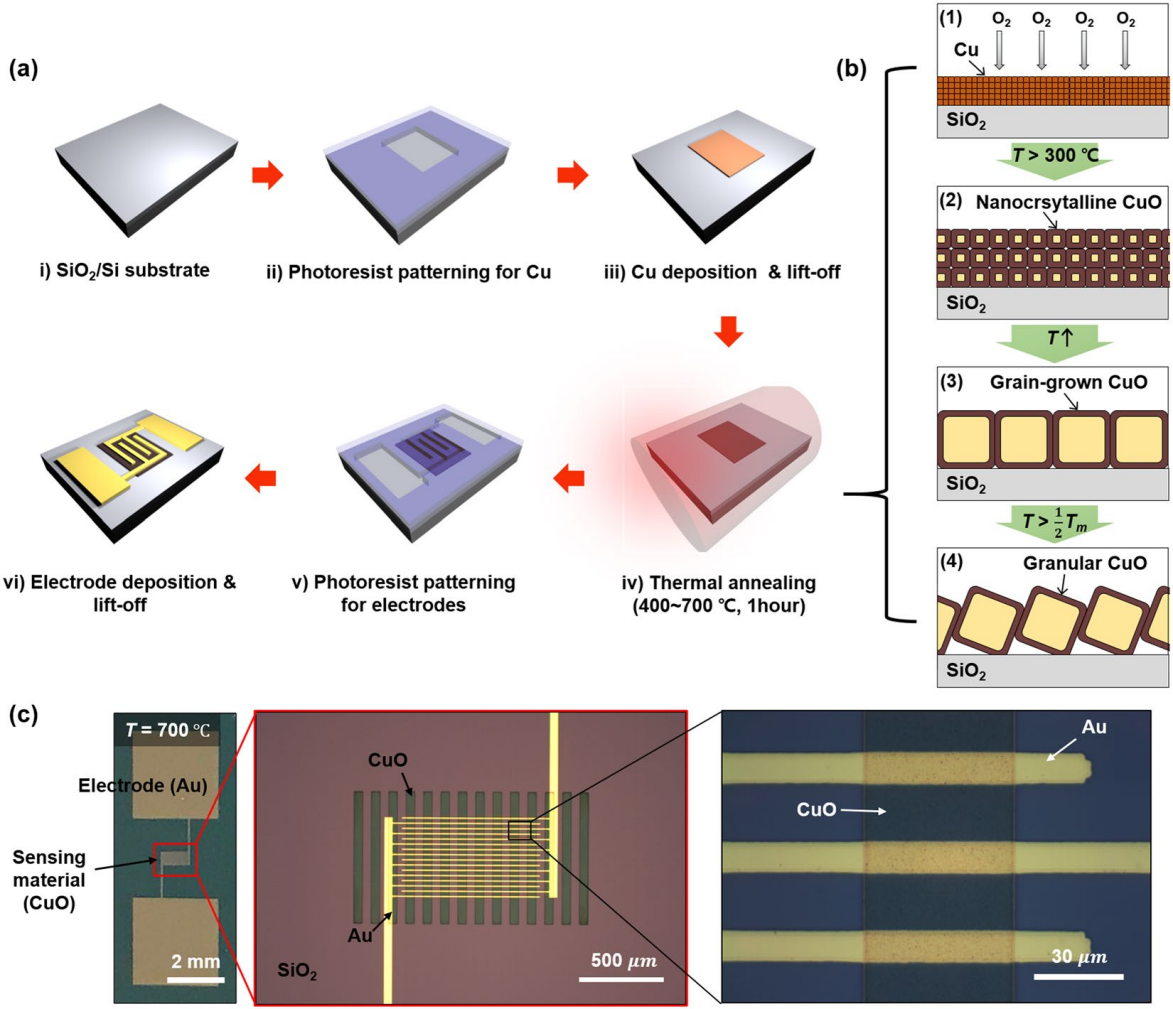
Demonstration of the CuO nanofilm photodetector. (**a**) Fabrication process of the CuO photodetector. (**b**) Cross-sectional schematic illustration of the process of grain structure control according to the increasing annealing temperature. (**c**) Photograph of the fabricated device annealed at 700 °C (left) and its magnified optical microscope images (middle, right).

**Figure 3:**
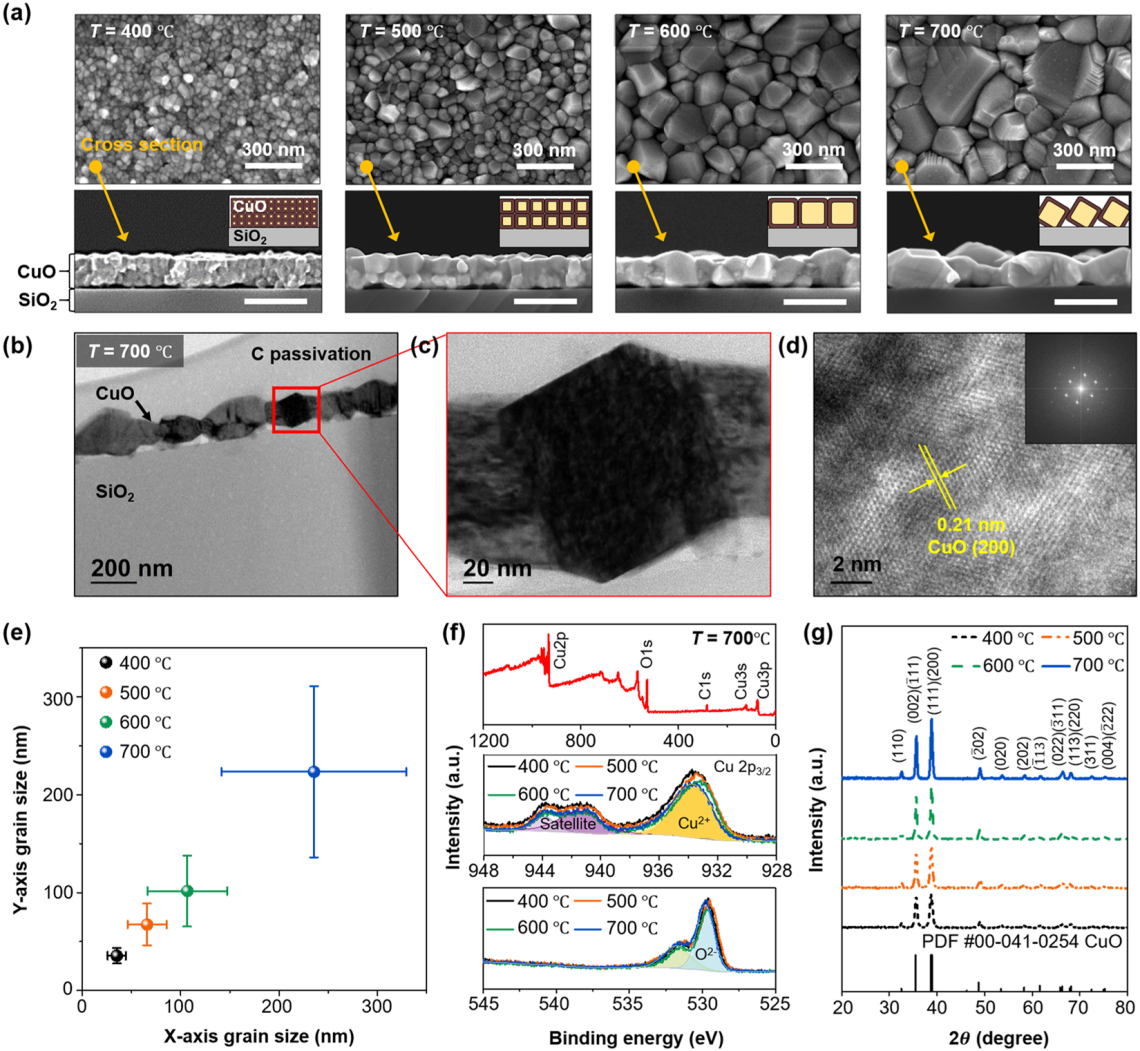
Material property of the fabricated CuO nanofilm. (**a**) Surface SEM images (upper panels) and cross-sectional SEM images (lower panels) of the CuO nanofilms annealed at 400, 500, 600, and 700 °C, respectively. Insets of lower panels show cross-sectional schematics of the CuO nanofilms. (**b**) TEM image, (**c**) Magnified TEM image, and (**d**) HRTEM image of the CuO nanofilm annealed at 700 °C. (**e**) Measured grain sizes, (**f**) XPS spectra results (top: wide-scan spectrum, middle: spectra for Cu 2 p, bottom: spectra for O1 s), and (**g**) XRD results of the nanofilms annealed at various annealing temperatures.

**Figure 4:**
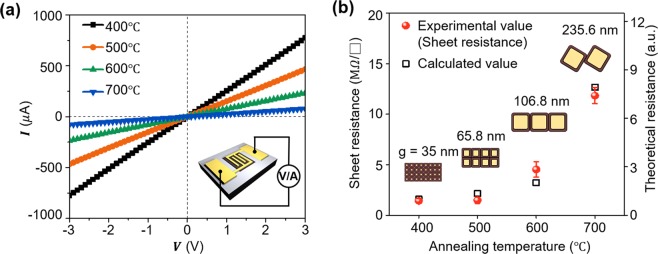
Electrical property of the fabricated CuO photodetectors. (**a**) *I*–*V* curves of the devices fabricated at various annealing temperatures. (**b**) Comparison between measured sheet resistance and theoretical resistance according to the annealing temperature.

To experimentally verify the proposed grain-structure model and to demonstrate the performance-enhanced CuO photodetector, we fabricated various types of CuO nanofilm photodetectors. In particular, we employed the metallic Cu nanofilm oxidation method for formation of the CuO nanofilms, not only to fabricate CuO grain structures with various shapes, from tens of nm to hundreds of nm, but also to keep other chemical states and material properties the same (Fig. 2). Figure 2(a,b) schematically shows the overall fabrication of the CuO photodetectors and the process how to control the grain structure, respectively. Fabrication starts with a silicon (Si) substrate on which a 1 μm-thick silicon dioxide (SiO_2_) layer is formed (Fig. 2(a,i)). Then, an 80 nm-thick Cu layer is deposited on the substrate by the conventional thermal physical vapor deposition (PVD) and lift-off methods (Fig. 2(a,ii,iii)). It should be noted that a strip-array configuration of Cu is patterned for the sensing film to alleviate the adhesion problem during the following high-temperature annealing process. The CuO nanofilm is formed and its grain structure is controlled through the thermal annealing process (Fig. 2(a,iv)). Conventionally, CuO is formed from Cu through oxidation at temperatures above 300 °C [2Cu + 1/2O_2_ → Cu_2_O, Cu_2_O + 1/2O_2_ → 2CuO]^46^. At such temperatures, the phase of Cu changes to CuO without grain growth because of insufficient thermal energy, resulting in a nanocrystalline structure (Fig. 2(b,1,2)). However, as the temperature further increases, the CuO nanocrystalline structure begins to grow its grain size. Especially, this grain growth process is drastically accelerated when the annealing temperature goes more than half of the melting temperature of the material, and then, the grain size can reach up to hundreds of nm from a few nm of the initial grain size (Fig. 2(b,3)). The neck-size control, which is the other important parameter in the proposed grain structure model, is also controlled by adjustment of the annealing temperature. When the annealing temperature is sufficiently high, the material starts to be wetting and form a granular CuO because of the surface tension^40^. Therefore, the granular CuO not only enhances its grain size, but also forms very small necks with adjacent grains (Fig. 2(b,4)). In our experiment, we obtained CuO nanofilms with various morphologies by annealing the Cu nanofilms at various temperatures of 400, 500, 600, and 700 °C for 1 hour in an air ambient with the heating rate of 10 °C/min and natural cooling. Finally, we formed 150 nm-thick gold (Au) electrodes on the oxidized CuO by the PVD and lift-off methods (Fig. 2(a,v,vi)). The fabricated CuO nanofilm photodetectors were investigated by optical microscope images (Fig. 2(c)). We confirmed that the device was successfully fabricated without any damage or adhesion problems, even after 700 °C high-temperature annealing.

## Results and Discussion

The grain structures of the annealed CuO nanofilms were investigated by scanning electron microscope (SEM), and their top-surface and cross-sectional images are shown in the upper and lower panels in Fig. 3(a), respectively (See Method section). From the top-surface SEM image, the CuO grain size of tens of nm is confirmed from the device with an annealing temperature of 400 °C (More information about top-surface SEM images and statistical histograms about grain-size variation in terms of the annealing temperature are provided in Supplementary Fig. S2). From the cross-sectional SEM images (lower panels in Fig. 3(a)), the film thickness is observed to be increased to 160 nm because of oxidation, within which CuO nanocrystals are stacked in multilayers. Increased grain size was obtained as the annealing temperature increased. As seen in the top-surface SEM images, the grain size significantly increased with increasing annealing temperature from 400 °C to 700 °C. We measured the x- and y-axis directional size of 200 units of grains in each specimen (Fig. 3(e)). The grain size increased continuously as the annealing temperature increased, and the x-axial sizes for annealing temperatures of 400 °C and 700 °C were ~35 nm and ~236 nm, respectively. Cross-sectional SEM images also show the drastic grain structure changes with respect to the annealing temperature. While the CuO nanofilms formed at 400  °C and 500  °C consisted of multiple layers of small grains, the nanofilms annealed at temperatures above 600  °C were composed of nearly single layers of large grains. Notably, the film annealed at 700  °C showed a reduced neck size. To ensure crystallinity in the single CuO grain, we performed transmission electron microscope (TEM) analysis of the CuO annealed at 700 °C (See Method section). As seen in the cross-sectional TEM image, the CuO film was composed of serially connected granular grains (Fig. 3(b,c)). Moreover, from high-resolution TEM (HRTEM) image (Fig. 3(d)) and diffraction patterns (Fig. 3(d) inset), we found that a single grain of CuO has high-quality single crystallinity. To confirm that crystallinity and chemical states of the fabricated CuO nanofilms were kept, even though they were annealed at various temperatures, and to objectively analyze the optoelectronic properties of the fabricated devices using the proposed model, we performed X-ray photoelectron spectroscopy (XPS) (Fig. 3(f)) and X-ray diffraction (XRD) analysis (Fig. 3(g)). From the wide-scan spectra, we confirmed that the CuO nanofilm consisted of only Cu, O, and C atoms. The spectra for Cu 2p showed a Cu 2p3/2 peak at ~933 eV and satellites consistent with CuO XPS spectra, where there is no difference according to the annealing temperature^47^. Also, O1s peaks at 530 eV were observed in all samples no matter what the annealing temperature was, which were in agreement with the peaks of CuO^48^. The XRD analysis results showed that the crystalline quality of the CuO nanofilm improved with increasing annealing temperature; however, all diffraction patterns showed high consistency with those of monoclinic CuO (PDF#00-041-0254) with no other phases (Fig. 3(g)). By the investigation of the CuO material properties, we confirmed that our fabrication method can control the grain structure precisely without any chemical disturbance.

**Figure 5:**
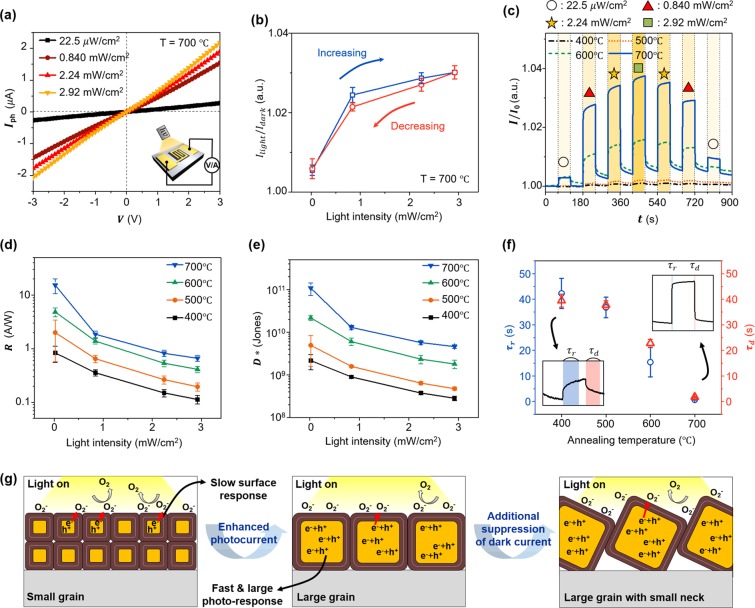
Optoelectronic properties of the CuO photodetectors. (**a**) *I*_*ph*_*-V* curves of the CuO photodetector annealed at 700 °C under various light intensities. (**b**) Measured *I*_*light*_/*I*_*dark*_ at 3 V bias of the device annealed at 700 °C under monotonically increase and decrease of the light intensity. (**c**) Time-dependent current normalized with initial value under different light intensities at 3 V bias. (**d**) Responsivity and (**e**) detectivity as a function of light intensity for devices fabricated at various annealing temperatures measured at 3 V bias. (**f**) Response time of the devices annealed at various annealing temperatures under the light intensity of 2.92 mW/cm^2^ with 3 V bias. (**g**) Schematic illustration of speculated operation principle and features in the CuO photodetector having different grain structures.

To characterize the electrical property of the fabricated photodetectors, the current-voltage (I–V) was measured in dark condition (Fig. 4(a)). The electrical measurement was performed using our customized probe station and semiconductor parameter analyzer (Keithley 2636B) at room temperature (See Method section). The gold (Au) electrical probes were contacted to Au pads of the fabricated device (inset in Fig. 4(a)). We confirmed that a lower dark current was achieved with a higher annealing temperature because of the increased volume fraction of the resistive core in the increased grain size. Using the proposed grain structure model, we calculated the electrical resistance of the fabricated CuO nanofilms and compared them with the experimental values (Fig. 4(b)) (See the Supplementary Information Table S1 for the information about all the calculated and measured results in detail). From the SEM images (Fig. 3(a)) and measured grain sizes (Fig. 3(e)), we modeled CuO nanofilms annealed at 400, 500, 600, and 700 °C into the simple electrical resistance system (calculations are described in detail in Fig. S3 and comparison of calculated and experimental values in Supplementary Information). We confirmed the calculated resistance increased with increasing annealing temperature, where the trend was in good agreement with that of the experimental results. Note that the lowest dark current was obtained from the CuO nanofilm annealed at 700 °C, which has the highest electrical resistance owing to the combined effects of the largest grain size and the reduced neck size. Based on the dark state result, we evaluated the optoelectronic properties of the photodetector annealed at 700 °C, which has the lowest dark current (the dark current of annealed CuO films at 400, 500, 600, and 700 °C was 774.5, 466.1, 233.7, and 79.05 *μA*, respectively, at 3 V) (Fig. 5(a,b)). Figure 5(a) shows the photocurrent (*I*_*ph*_ = *I*_*light*_ − *I*_*dark*_) as a function of the bias voltage under various intensities of white light ranging from 22.5 μW/cm^2^ to 2.92 mW/cm^2^. For the characterization of the proposed visible light photodetector, we used a commercial visible light halogen lamp (halogen-Fok-100W, Fiber Optic Korea Co., LTD.), which has a peak wavelength at 650 nm (The wavelength spectrum of the white light is provided in Supplementary Fig. S4). The *I*_*ph*_ showed almost a linear relationship with the bias voltage and increased with higher light intensity. We also investigated the photosensitivity (defined as *I*_*ph*_/*I*_*dark*_ × 100) under the condition of monotonic increase and decrease in light intensity (Fig. 5(b)). Although a slight hysteresis was observed, which can probably be attributed to the dark current that has not fully recovered to its initial state due to the slow oxygen desorption reaction, the photodetector showed a stable and reversible photo-sensing property. To compare the performance of the photodetector annealed at 700 °C with other photodetectors annealed at 400, 500, and 600 °C, we measured the time-dependent photo-response under various light intensities at a bias voltage of 3 V (Fig. 5(c)). A larger photocurrent was observed with a higher annealing temperature (The *I*_*ph*_-*V* curves according to the annealing temperature are shown in Supplementary Fig. S5). To investigate the performance improvement according to the annealing temperature, we compared the responsivity and detectivity as a function of the light intensity at 3 V bias (Fig. 5(d,e)). The responsivity (*R*) represents the photocurrent per incident optical power, which is calculated as *R* = *I*_*ph*_/*P*_*in*_ *S*, where *P*_*in*_ is the light intensity, and *S* is the illuminated area (Fig. 5(d))^17^. The increased *I*_*ph*_ with higher annealing temperature contributed to the increased *R*, which led to the high responsivity from 15.3 to 0.662 A/W obtained for the device annealed at 700 °C within the light intensity range of 0.0225 to 2.92 mW/cm^2^. In particular, the responsivity of 15.3 A/W at the light intensity of 0.0225 mW/cm^2^ was 18 times higher than the value of 0.833 A/W obtained for the device annealed at 400 °C. In addition, another important figure of merit, the specific detectivity (D*) associated with the weakest detectable optical signal was obtained by calculating D* = *R/(2eI*_*dark*_*/S)*^1/2^ (Fig. 5(e))^17^. Because high detectivity is realized by high responsivity and low dark current, the photodetector annealed at 700 °C exhibited the highest detectivity, where the maximum value of 1.08 × 10^11^ Jones was 50 times higher than the value of 2.16 × 10^9^ Jones obtained for the photodetector annealed at 400 °C at a light intensity of 0.0225 mW/cm^2^. The reduced responsivity and detectivity with the increased light intensity can be explained by the enhanced combination of excitons due to the increased carrier concentration^19^. Figure 5(f) shows the response time obtained from the photo-switching result with the light-intensity of 2.92 mW/cm^2^. The rise time (*τ*_*r*_) is defined as the time required to increase from 10% to 90% of the maximum current after the light is turned on, and the decay time (*τ*_*d*_) is defined as the time to decay from 90% to 10% of the maximum current after the light is turned off^40^. In comparison to the rise time and decay time of 42.3 s and 39.4 s, respectively, from the device annealed at 400 °C, we achieved greatly reduced rise time and decay time of 0.7 s and 1.8 s, respectively, from the device annealed at 700 °C (rise/decay time of device annealed at 400, 500, 600, and 700 °C was 42.3/39.4, 36.9/37.8, 15.4/22.8, and 0.7/1.8 s, respectively). These improvements according to the annealing temperature can be explained by Fig. 5(g). As shown in Fig. 1(c,ii,iii), the photocurrent is generated by two mechanisms: photo-carrier generation at the resistive core and oxygen adsorption response at the hole accumulation layer. In the case of a small grain, the slow surface reaction is more dominant because of the large volume fraction of the shell, whereas for a large grain, fast and large photo-carrier generation is dominant due to the increased volume fraction of the core. As a result, a larger-grain-based CuO photodetector shows a larger photocurrent and faster response speed than a smaller-grain-based photodetector, which results in improved performance. Meanwhile, as the neck size becomes smaller, there is an additional dark current suppression effect, which contributes to the performance enhancement (Fig. 1(e)). In this work, our CuO photodetector annealed at 700 °C showed high responsivity and detectivity as well as fast response time owing to the large grain and small neck, which were superior to those of previously reported CuO film photodetectors, and even higher than some other types of CuO nanostructure (0D, 1D) photodetectors. We also studied about the relationship between responsivity and the device channel length, and we verified that the responsivity of the CuO device can be changed by the channel length because the carrier transit time, which determines the photo-current depends on the channel-length (See Supplementary Information Fig. S6)^49^. Furthermore, our film-based photodetector is more desirable for practical usage because of its higher process controllability and stability than other types of nanostructure based photodetectors (See Supplementary Table S2).

**Figure 6:**
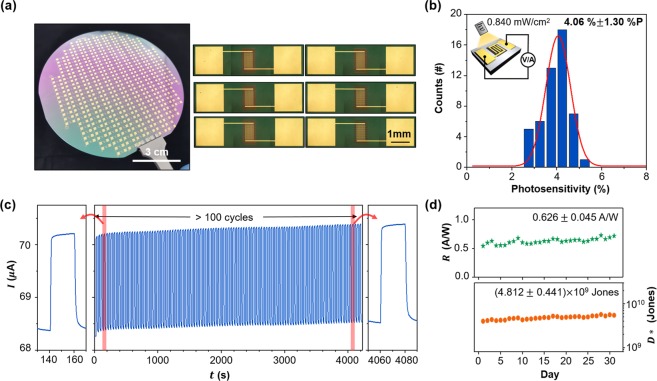
Large-area (4-inch) uniformity and device reliability of the fabricated CuO photodetector. (**a**) Photograph of the fabricated photodetector array (left panel) and magnified optical microscope images of the devices (right panels). (**b**) Photosensitivity histogram of 50 devices measured under the light intensity of 0.840 mW/cm^2^ at 3 V bias. (**c**) Time-dependent current under repeated photo-switching with the light intensity of 2.92 mW/cm^2^ at 3 V bias. (**d**) Stability test result of the photodetector measured under the light intensity of 2.92 mW/cm^2^ with 3 V bias for 31 days.

To confirm the practicality of the proposed device, we demonstrated a 4-inch-scale CuO photodetector array annealed at 700 °C, which had the highest performance, and confirmed its durability (Fig. 6). From the optical image and microscope images, we confirmed that the array was stably fabricated (left panel in Fig. 6(a)), and the devices were fabricated uniformly over the entire area of the 4-inch Si wafer (right panels in Fig. 6(a)). To verify the performance uniformity, we measured the photosensitivity of 50 devices located sparsely on the 4-inch wafer. The devices showed highly uniform photosensitivity with the average of 4.06% and the standard deviation of 1.30% P (point) (Fig. 6(b)). This result indicates that CuO is compatible with the conventional semiconductor process and is suitable for the large-area fabrication. To test the device durability for practical applications, we performed repeated photo-switching operation over 100 cycles at a 3 V bias and confirmed stable photo-response (Fig. 6(c)). Moreover, to evaluate the long-term stability, we measured the device performance for 31 days. The device maintained its responsivity and detectivity for 31 days owing to the material stability of CuO annealed at 700 °C (Fig. 6(d)). The CuO photodetector fabricated by high-temperature annealing not only showed enhanced performance due to the proper grain structure but also high productivity and device stability, which is suitable for high-performance and practical optoelectronic applications.

## Conclusion

In conclusion, we developed a grain-structure model of a CuO visible-light photodetector and demonstrated its high performance by controlling the grain structure. To investigate the effect of grain structure and thereby enhance the optoelectronic characteristics, we fabricated polycrystalline CuO nanofilms with various grain and neck sizes by controlling the annealing temperature. The CuO nanofilm annealed at 700 °C exhibited the largest grain size of ~236 nm and the reduced neck size; it showed the highest responsivity of 15.3 A/W and detectivity of 1.08 × 10^11^ Jones, which are 18 and 50 times higher than those of the smallest grain nanofilm annealed at 400 °C. In addition, the fastest response times of 0.682 s (rise) and 1.77 s (decay) were obtained from the 700 °C annealed CuO photodetector. This result was explained by the influence of the increased grain size, namely the increased volume fraction of the resistive core and the reduced neck size, leading to the suppressed dark current and enhanced photocurrent generation. By fabricating a large-area photodetector array, we confirmed the high productivity and performance uniformity of the CuO photodetector. The fabricated CuO photodetector exhibited excellent durability over 100 cycles of operation, and the performance was not degraded for 31 days. The proposed method will be a guideline for the design and fabrication of high-performance visible-light photodetectors, and it opens up a new way to realize of next-generation optoelectronic devices.

## Methods

### Material characterization

SEM and TEM images were obtained using FEI-Sirion and TEM, respectively. The chemical states were investigated by Thermo VG Scientific Sigma Probe X-ray photoelectron spectroscopy. Rigaku D/MAX-2500 X-ray diffractometer (CuK_α_ 1.5406 Å radiation) was used for investigating the crystalline quality.

### Device characterization

The *I*–*V* characteristics was measured with probe station connecting with Keithley 2636B sourcemeter. For confirming optoelectronic characteristics, the devices were illuminated with white light halogen lamp where the light intensities were measured with a Thorlabs PM 100d power meter.

## Supplementary information

Supplementary Information
