# Supplementary material for: High-Performance Copper Oxide Visible-Light Photodetector via Grain-Structure Model
Source: Sci Rep. 2019 May 14;9:7334. doi: 10.1038/s41598-019-43667-9 (PMC6517403; doi:10.1038/s41598-019-43667-9)
Supplement: Supplementary file 1 — Supplementary Information [file 41598_2019_43667_MOESM1_ESM.docx]

Supplementary Information

High-Performance Copper Oxide Visible-Light Photodetector via Grain-Structure Model

*Hyeon-Joo Song^1, †^, Min-Ho Seo^1, 2, †^, Kwang-Wook Choi^1^, Min-Seung Jo^1^, Jae-Young Yoo^1^, and Jun-Bo Yoon^1, *^*

^1^ School of Electrical Engineering, Korea Advanced Institute of Science and Technology (KAIST), 291 Daehak-ro, Yuseong-gu, Daejeon 34141, Republic of Korea

^2^ Information & Electronics Research Institute Korea Advanced Institute of Science and Technology (KAIST), 291, Daehak-ro, Yuseong-gu, Daejeon 34141, Republic of Korea

* [jbyoon@kaist.ac.kr](mailto:jbyoon@kaist.ac.kr)

^†^ These authors contributed equally to this work.

**Keywords:** visible light, photodetectors, copper oxide, grain structures, annealing


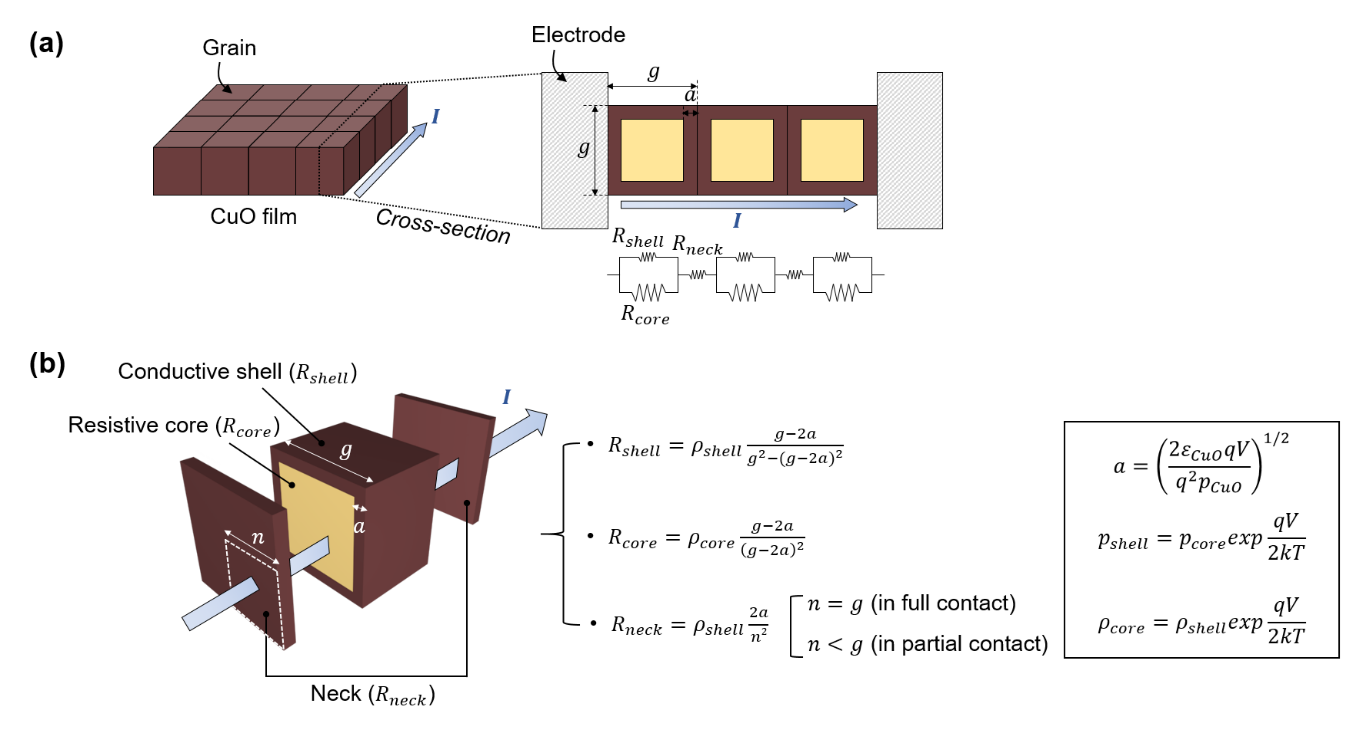


**Figure S1**. Grain-structure model for the CuO film photodetector (a) Schematic of simplified polycrystalline CuO film consisted of grain model and its cross-sectional view, (b) simple cube grain model ($g$: grain size, $a$: hole accumulation layer depth) [1]

**Concept and calculation method**

We represent polycrystalline CuO film with a matrix of simple grain models. Each grain model consists of three parts: resistive core, conductive shell and two necks (i.e. the parts of the conductive region in contact with the adjacent grains along the direction of current flow). The resistance of the grain model is calculated as parallel connection of resistive core (*R_core_*) and conductive shell (*R_shell_*) and serial connection of two necks (*R_neck_*) (Fig. S1 (a, b)). Each resistance can be calculated as below in cubic grain model^1^:

$R_{shell}=\rho_{shell}\frac{g-2a}{g^{2}-{(g-2a)}^{2}}$ (S1)

$R_{core}=\rho_{core}\frac{g-2a}{\left( g-2a \right)^{2}}$ (S2)

$R_{neck}=\rho_{shell}\frac{2a}{n^{2}}$ (S3)

where $g$, $a$ and $n$ are grain size, hole accumulation layer depth and neck size, respectively, and $\rho_{shell}$ and $\rho_{core}$ are the resistivity values of hole accumulation layer and resistive core, respectively. Hole accumulation layer depth is calculated as $a=\left( \frac{2\varepsilon_{CuO}qV}{q^{2}p_{CuO}} \right)^{1/2}$, where $\varepsilon_{CuO}$ is the permittivity of CuO ($\approx$25$\times$8.854$\times$10^-12^ F/m), *q* is the electron charge (=1.6$\times$10^-19^ C), *qV* is the height of the potential barrier formed by oxygen adsorption ($\approx0.59 eV)$, $p_{CuO}$is the majority carrier concentration of CuO ($\approx$10^19^ cm^-3^ at room temperature)^2^. The neck size ($n$) is equal to the grain size ($g$) when the grain is connected with the adjacent grain in full contact, but is smaller than $g$ if the grain is in partial contact with the adjacent grain. The relation of hole concentration in shell ($p_{shell}$) and core ($p_{core}$) is $p_{shell}=p_{core}exp\frac{qV}{2kT}$, which leads to the relation of resistivity as $\rho_{core}=\rho_{shell}exp\frac{qV}{2kT}$ ^3^.

To predict the effect of grain size and neck size on dark current in CuO film, the resistance of film is calculated using the grain model. We calculated the film resistance by serial connections of grains if the array of grains is parallel to the direction of current flow, and by parallel connections if it is perpendicular under the same conditions of film thickness and area. Note that we applied different calculation method for two cases: (i) $g$ is smaller than film thickness, (ii) $g$ is larger than film thickness. For the first case, we assumed that the film is composed of several layers of cube grain models, where the average number of layers can be calculated by dividing the film thickness by the grain size. Thus, the film is regarded as the parallel connections of each layer in vertical direction. On the other hand, for the second case, the film is regarded as the monolayer of grain models and the height of the grain model is considered to be equal to the thickness of the film. Based on the calculation method, the effect of grain size on dark current and the volume fraction of core are shown in Fig. 1(d) by varying the grain size ($g$) in the grain model. Also, based on the film which is the monolayer of large grains, we predicted the effect of decreasing neck size on dark current, considering the effective contacting area (Fig. 1(e)).


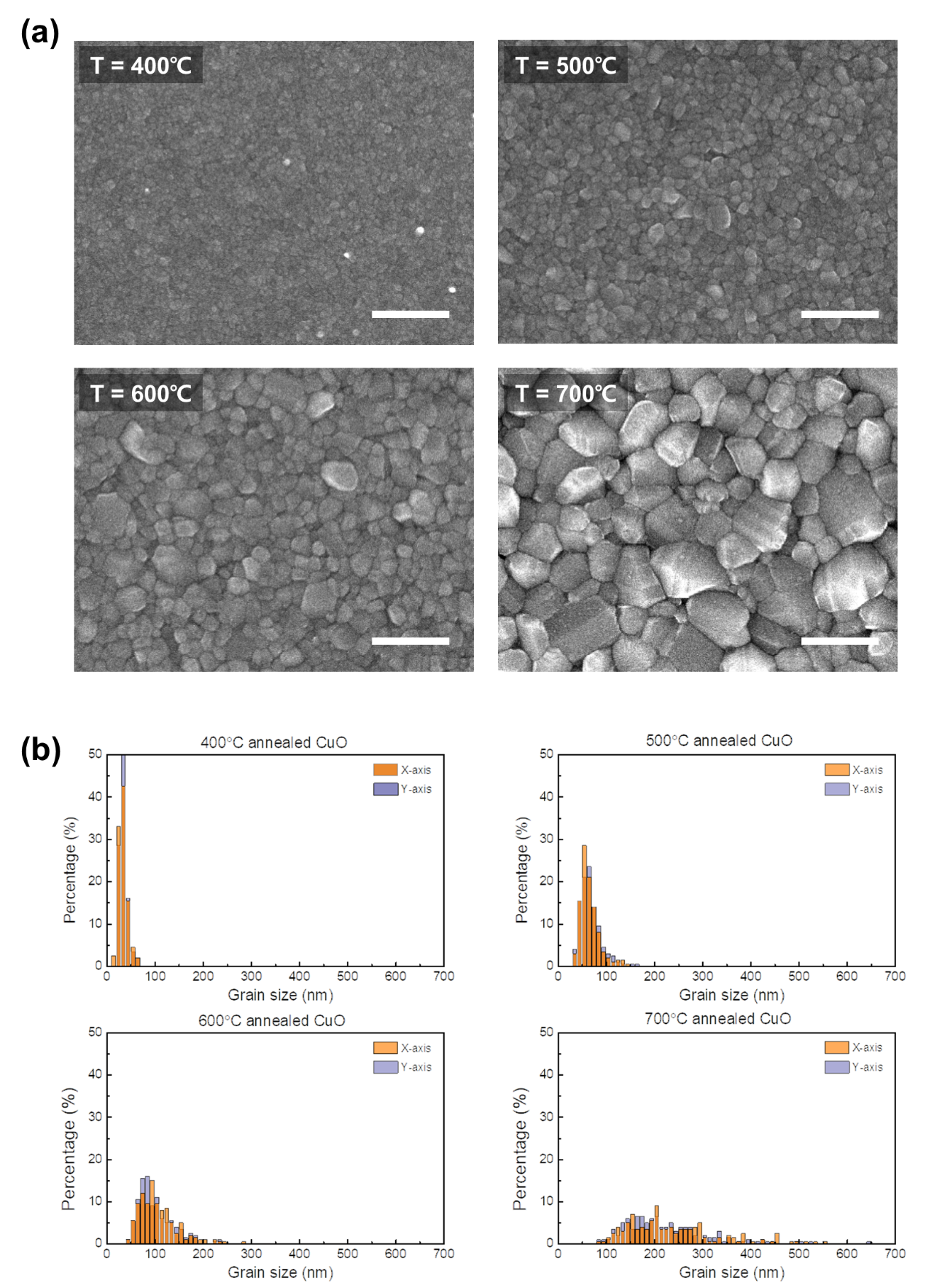


**Figure S3.** (a) SEM images of the CuO films annealed at 400, 500, 600, and 700 $\mathbf{℃}$. Scale bar, 500 nm. (b) histograms of grain-size distribution, measured along to w-/y-axis of the CuO annealed at at 400, 500, 600, and 700 $℃$.

**Table S1**. Information about all the calculated and measured results. $a$and $g$ are hole accumulation layer depth and grain size, respectively, and $\rho_{core}$ and $\rho_{shell}$ are the resistivity values of resistive core and hole accumulation layer, respectively.

|  | Theoretical value | | | |
| --- | --- | --- | --- | --- |
| a (nm) | 12.7 [2] | | | |
| $\rho_{core}/\rho_{shell}$ (a.u.) | 84639.3 [3] | | | |
| *T* ($℃$) | Measured value | | Calculated value | |
|  | $g$ (nm) | Sheet resistance ($M\Omega$/□) | Neck size (nm) | Normalized resistance (a.u.) |
| 400 | 35.0 | 1.43 | 35 | 1.00 |
| 500 | 65.8 | 1.49 | 65.8 | 1.34 |
| 600 | 106.8 | 4.53 | 106.8 | 2.01 |
| 700 | 235.6 | 11.83 | 30* | 7.92 |

* Note that the reduced neck size (n) of 30 nm was measured from the experimentally 700 $℃$ annealed CuO film (Fig. 3(b) in manuscript).


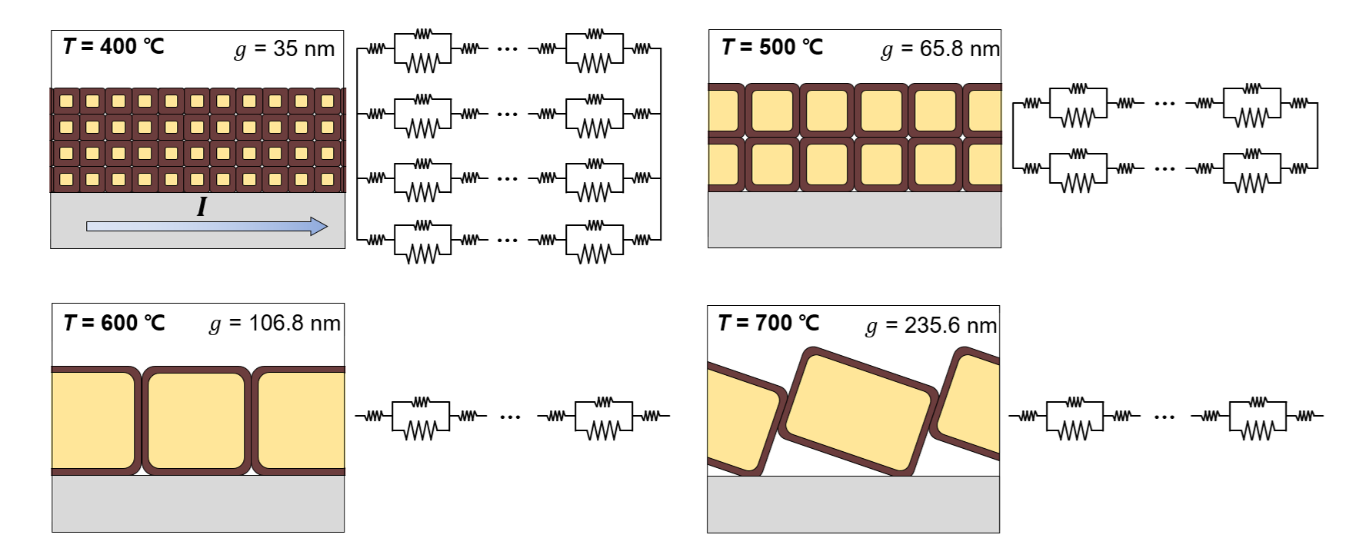


**Figure S2**. Cross sectional schematic illustration of the fabricated CuO photodetector and corresponding equivalent resistance circuits according to the annealing temperature (400, 500, 600, and 700 $\mathbf{℃}$)

**Comparison of calculated and experimental values**

To confirm the agreement between calculated resistance and experimental sheet resistance, we first calculated sheet resistance using the resistance obtained from the measured current at -3 V bias in Fig. 4(a) and the geometric parameters of the device. More specifically, since we fabricated the device with strip-array configuration based sensing material and interdigitated electrodes, the sheet resistance was calculated by the following equation:

$$R_{s}=(N_{IDE}-1)N_{strip}\times\frac{w}{l}\times R$$

where the $R_{s}$, $w$, $l$ and $R$ are the sheet resistance, the width, the length of each conductive channel, and the measured resistance, respectively, and $N_{IDE}$ and $N_{strip}$ are the number of interdigitated electrodes and strips, respectively. Since our device has the geometric parameters of $w$, $l$, $N_{IDE}$ and $N_{strip}$ are 50 $\mu m$, 20 $\mu m$, 16, and 8, respectively, therefore, the calculated sheet resistance of device annealed at 400, 500, 600, and 700 $℃$ was 1.16, 1.93, 3.85, and 11.4 $M\Omega$/□, respectively.

We also calculated the thoeretical values using the measured grain sizes of the films annealed at different temperatures (Fig. 4(b)). Under the same film thickness (160 nm) condition, we applied the multiple layers of grains to the resistance calculation of films annealed at 400, 500 $\mathbf{℃}$ and 600 $\mathbf{℃}$ because these have the grain sizes of 35, 65.8, and 106.8 nm, respectively, which are smaller than the film thickness. The average number of layers are obtained by dividing the film thickness by measured grain sizes and corresponds to 4.6, 2.4, and 1.5 for 400, 500, and 600 $\mathbf{℃}$, respectively. The film annealed at 700 $\mathbf{℃}$ is considered as the monolayer of grains due to its large grain size of 235.6 nm (Fig. S2). Moreover, 700 $\mathbf{℃}$ annealed film has reduced neck size as shown in Fig. 3(a), thus the resistance of the film was calculated with the reduced neck size ($n$) of 30 nm.


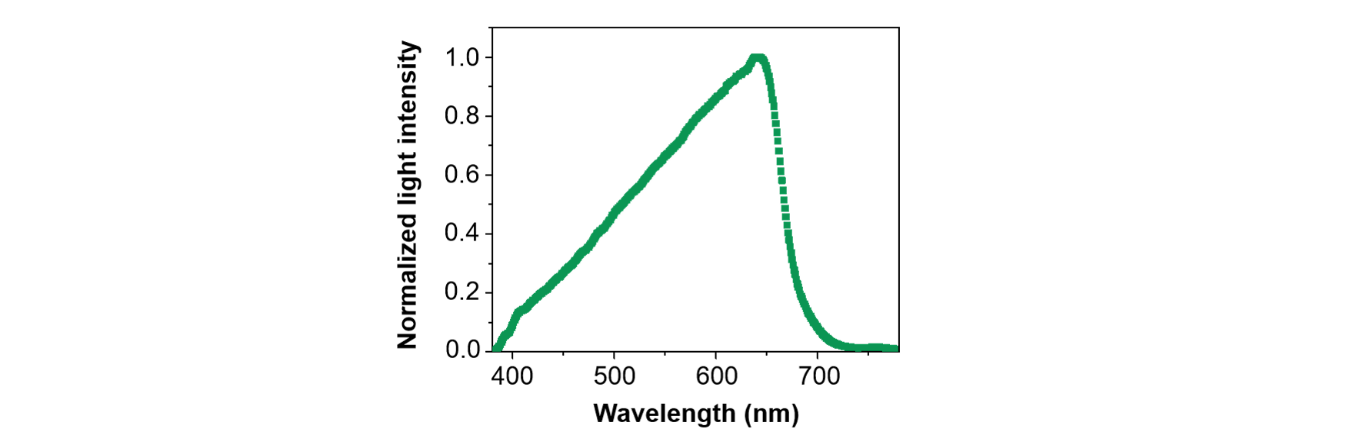


**Figure S4.** Normalized spectra of white light used for illuminating the photodetector


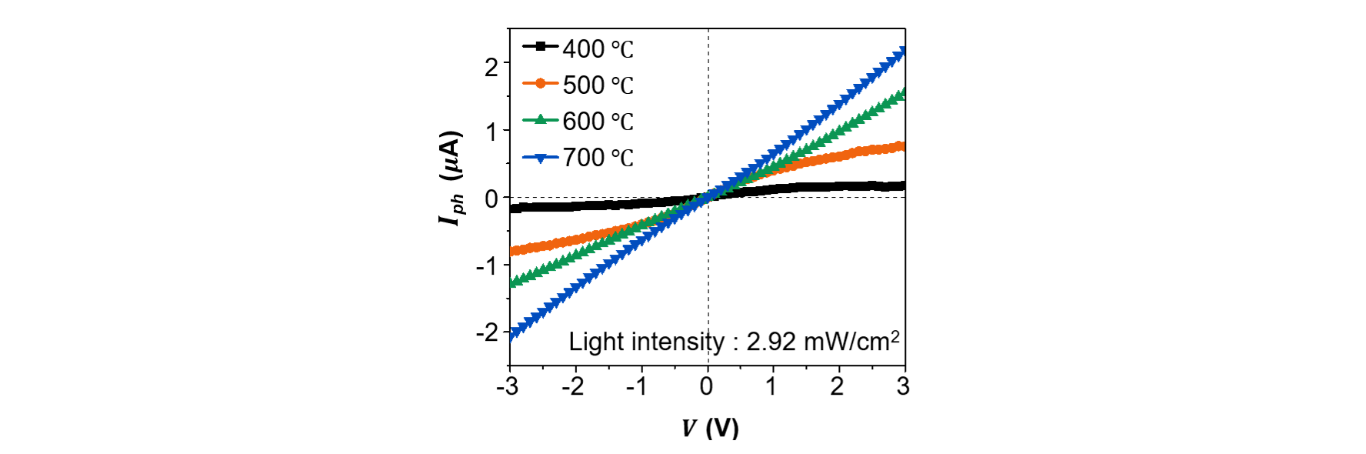


**Figure S5.** *I_ph_*-*V* curves of the CuO photodetectors fabricated with different annealing temperatures under irradiance of 2.92 mW/cm^2^


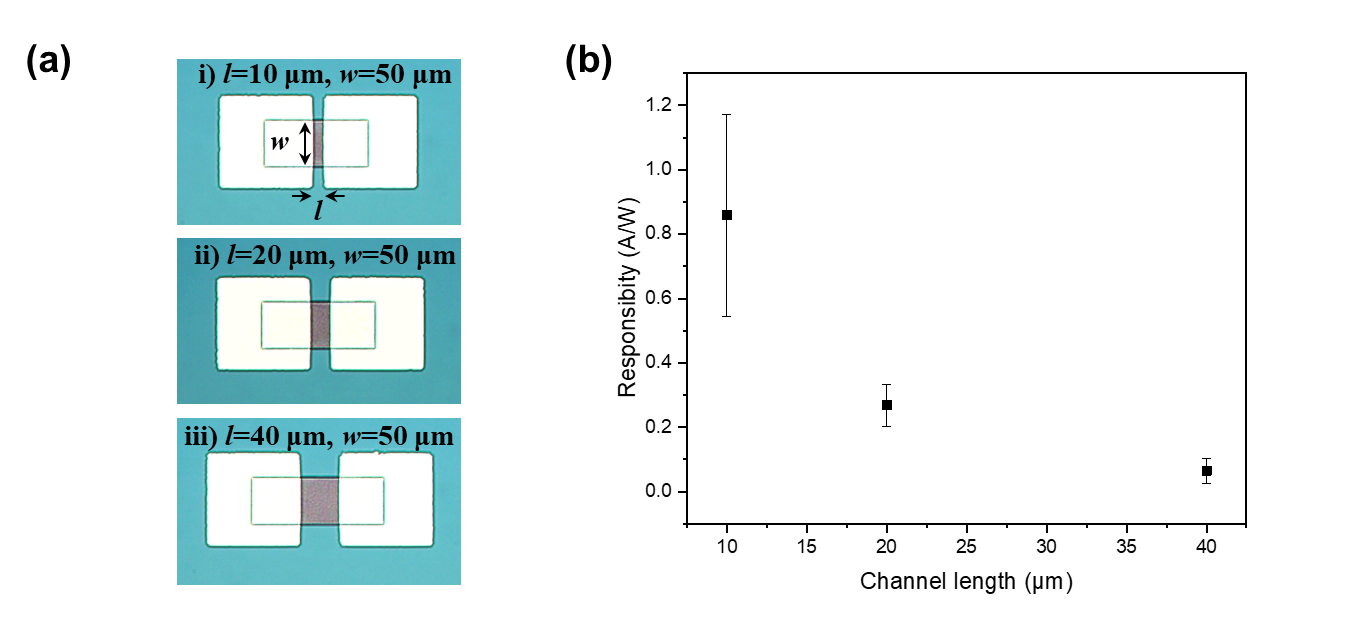


Figure S6. (a) Optical microscope images of the fabricated devices having different channel length (*l*=10, 20, and 40 μm). The width (*w*) is 50 μm. (b) Calculated responsivity with respect to the channel length (light intensity=2.92 mW/cm^2^).

**Table S2**. Comparison of the characteristic parameters with previously reported visible light photodetectors

**
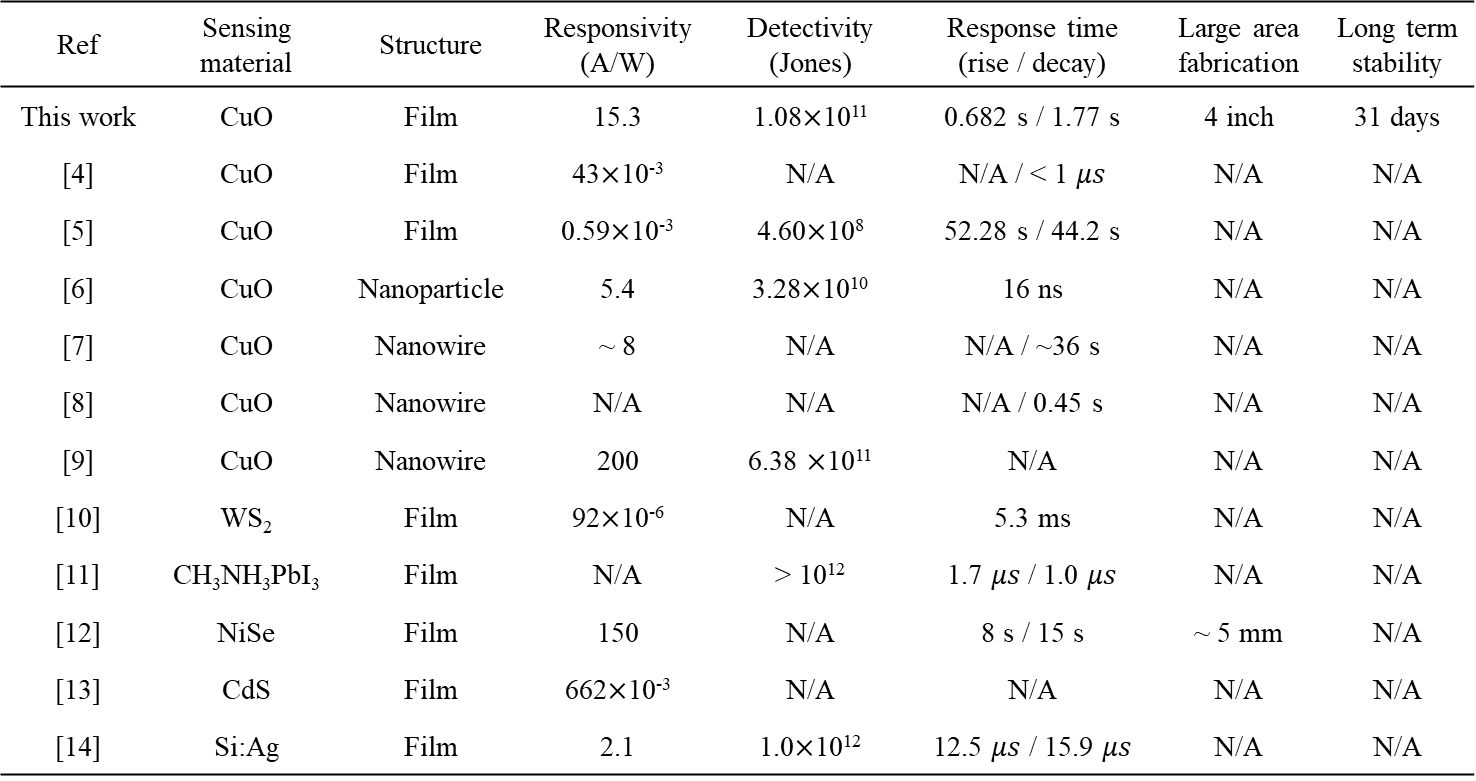
**

**References**

1. Barsan, N., Simion, C., Heine, T., Pokhrel, S. & Weimar, U. Modeling of sensing and transduction for p-type semiconducting metal oxide based gas sensors. *J. Electroceram* **25,** 11-19 (2010).
2. Choi, S.-W., Katoch, A., Kim, J.-H. & Kim, S. S. A novel approach to improving oxidizing-gas sensing ability of p-CuO nanowires using biased radial modulation of a hole-accumulation layer. *J. Mater. Chem. C* **2,** 8911-8917 (2014).
3. Zhang, J., Qin, Z., Zeng, D. & Xie, C. Metal-oxide-semiconductor based gas sensors: screening, preparation, and integration. *Phys. Chem. Chem. Phys.* **19,** 6313-6329, (2017).
4. Hong, M.-J., Lin, Y.-C., Chao, L.-C., Lin, P.-H. & Huang, B.-R. Cupric and cuprous oxide by reactive ion beam sputter deposition and the photosensing properties of cupric oxide metal–semiconductor–metal Schottky photodiodes. *Appl. Surf. Sci.* **346,** 18-23 (2015).
5. Raghavendra, P. V., Bhat, J. S. & Deshpande, N. G. Visible light sensitive cupric oxide metal-semiconductor-metal photodetectors. *Superlattice. Microstruct.* **113,** 754-760 (2018).
6. Jana, R. *et al.* Improving performance of device made up of CuO nanoparticles synthesized by hydrothermal over the reflux method. *Appl. Surf. Sci.* **452,** 155-164 (2018).
7. Hansen, B. J. *et al.* Transport, analyte detection, and opto-electronic response of p-type CuO nanowires. *J. Phys. Chem. C* **114,** 2440-2447 (2010).
8. Ko, Y. H., Nagaraju, G., Lee, S. H. & Yu, J. S. Facile preparation and optoelectronic properties of CuO nanowires for violet light sensing. *Mater. Lett.* **117,** 217-220 (2014).
9. Luo, L.-B. *et al.* One-dimensional CuO nanowire: synthesis, electrical, and optoelectronic devices application. *Nanoscale Res. Lett.* **9,** 637 (2014).
10. Néstor, P.-L. *et al.* Photosensor device based on few‐layered WS2 films. *Adv. Func. Mater.* **23,** 5511-5517 (2013).
11. Lin, Q., Armin, A., Lyons, D. M., Burn, P. L., & Meredith, P. Low Noise, IR‐Blind Organohalide Perovskite Photodiodes for Visible Light Detection and Imaging. *Adv. Mater.* **27,** 2060-2064 (2015).
12. Cai, C. *et al.* Epitaxial Growth of Large‐Grain NiSe Films by Solid‐State Reaction for High‐Responsivity Photodetector Arrays. *Adv. Mater.* **29,** 1606180 (2017).
13. Zhao, Y. *et al.* Size controllable preparation of sphere-based monolayer CdS thin films for white-light photodetectors. *Ceram Int.* **44,** 2407-2412 (2018).
14. Qiu, X. *et al.* Trap Assisted Bulk Silicon Photodetector with High Photoconductive Gain, Low Noise, and Fast Response by Ag Hyperdoping. *Adv. Opt. Mater.* **6,** 1700638 (2018).
